# Pressurized intraperitoneal aerosol chemotherapy, reasons for interrupting treatment: a systematic review of the literature

**DOI:** 10.1515/pp-2023-0004

**Published:** 2023-04-19

**Authors:** Anne-Cecile Ezanno, Brice Malgras, Marc Pocard

**Affiliations:** Department of digestive surgery , Begin Military Teaching Hospital , Saint Mandé , France; French Military health Service Academy, Ecole du Val de Grâce, Paris, France; Department of digestive surgery, La Pitié Salpétrière Hospital, Paris, France; INSERM, U965 Cart unit, Paris, France

**Keywords:** colon cancer, gastric cancer, mesothelioma, peritoneal metastasis, PIPAC, pressurized intraperitoneal aerosol chemotherapy, safety process

## Abstract

**Objectives:**

Pressurized intraperitoneal aerosol chemotherapy (PIPAC) gives encouraging results in the treatment of peritoneal metastasis (PM). The current recommendations require at least 3 sessions of PIPAC. However, some patients do not complete the full treatment course and stop after only 1 or 2 procedures, hence the limited benefit. A literature review was performed, with search terms including “PIPAC” and “pressurised intraperitoneal aerosol chemotherapy.”

**Content:**

Only articles describing the causes for premature termination of the PIPAC treatment were analysed. The systematic search identified 26 published clinical articles related to PIPAC and reporting causes for stopping PIPAC.

**Summary:**

The series range from 11 to 144 patients, with a total of 1352 patients treated with PIPAC for various tumours. A total of 3088 PIPAC treatments were performed. The median number of PIPAC treatments per patient was 2.1, the median PCI score at the time of the first PIPAC was 19 and the number of patients who did not complete the recommended 3 sessions of PIPAC was 714 (52.8%). Disease progression was the main reason for early termination of the PIPAC treatment (49.1%). The other causes were death, patients’ wishes, adverse events, conversion to curative cytoreductive surgery and other medical reasons (embolism, pulmonary infection, etc…).

**Outlook:**

Further investigations are necessary to better understand the causes for interrupting PIPAC treatment and also improving the selection of patients who are most likely to benefit from PIPAC.

## Introduction

Peritoneal metastasis (PM) is associated with a poor prognosis in the absence of effective multimodal therapeutic approaches. Pressurized intraperitoneal aerosol chemotherapy (PIPAC) has emerged with encouraging results in the treatment of PM [1], [2], [3] in the last ten years. The current recommendations require at least 3 PIPAC procedures planned every 4–6 weeks [4, 5]. Unfortunately, many patients do not complete the 3 planned procedures and stop PIPAC prematurely. There are many reasons for discontinuing after only 1 or 2 procedures, with the main reason being the progression of the oncological disease [6, 7]. We therefore aimed to investigate reasons for stopping PIPAC, to better select patients.

The aim of the present study is to propose a systematic review of the literature in order to evaluate the number and reasons for discontinuing PIPAC procedures.

## Materials and methods

**Figure 1:**
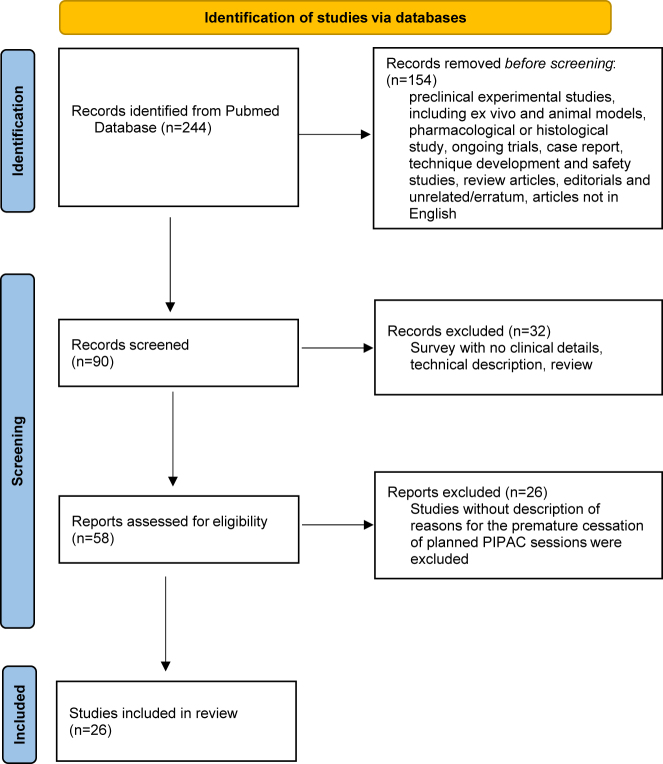
Flow-chart.

A literature review was performed according to the Preferred Reporting Items for Systematic Reviews and Meta-Analyses (PRISMA) guidelines [8]. A literature search was performed by using the Medline database (via PubMed). This first search step was performed without any language restriction. Search terms included “PIPAC” and “pressurized intraperitoneal aerosol chemotherapy.” After removing duplicates, a total of 244 articles were identified up to August 1, 2022. Only articles published in English were analysed in the second steep. The following publications were excluded: case reports of 5 patients or fewer, preclinical experimental studies, including *ex vivo* and animal models, pharmacological or histological study, ongoing trials, case reports, technique development and safety studies, review articles, editorials and unrelated/erratum (flowchart, Figure 1). We only analysed articles that described the reasons for discontinuing PIPAC treatment.

For each article, when available, the following items were recorded for each study: authors, title, year of publication, primary tumour origin, number of patients, details of patients (median age of patients), previous chemotherapy and number of previous lines of systemic chemotherapy, total number of PIPAC procedures, details of number of PIPAC and protocol (bimodal treatment or not), median number of PIPAC sessions per patient, details on the surgical intervention (rate of non-access, peritoneal carcinomatosis index [PCI], ascites at PIPAC#1 and intraoperative complications). Postoperative outcome measures included postoperative surgical complications (morbidity), mortality, and reasons for stopping PIPAC before three sessions had been completed, which were separated into 6 groups: non-access, conversion to curative cytoreductive surgery, tumour progression intra- or extraperitoneal, patient wishes, postoperative complications/adverse events (compromising other PIPAC), death or other medical reasons (pulmonary embolism, infection, myocardial infarction, protocol violation or other cancer).

### Statistical analysis

Small sample size and heterogeneity in the original data did not allow for proper statistical meta-analysis. Microsoft Excel version 16.60 and R version 4.1.3 was used respectively for data collection and statistical analysis. Continuous data are presented using descriptive statistics mean ± standard deviation or median (25th; 75th percentiles). Categorical data are presented using frequencies and percentages. Quantitative parameters were compared between groups using the Student’s t-test or Mann–Whitney test when normality was rejected. Qualitative parameters were compared between groups using the Chi-square test or Fisher exact test, as appropriate. A threshold of 5 % was used to define the significance of the statistical tests.

## Results

In the literature, a limited number of studies include the reasons for the early discontinuation of PIPAC procedures especially after 1 or 2 sessions. The systematic search identified 26 published clinical articles related to PIPAC with inclusion criteria (Figure 1, Flow chart).

The studies included 11 to 183 patients, with the number of reported PIPAC sessions of 17–517. The origin of the peritoneal metastasis of the overall group was colorectal (5; 19.2 %), biliary tract and pancreatic (3; 11.6 %), gastric (5; 19.2 %), ovarian (1; 3.8 %), mesothelioma (1; 3.8 %), gynaecological (1; 3.8 %) and various (10; 38.6 %). Studies and patient characteristics are summarised in Table 1. A total of 3,088 PIPAC procedures were performed in 1,278 patients.

**Table 1:** Characteristics of PIPAC studies.

Reference	Primary tumour	Numbers of patients, n	Median age, years	Primary no abdominal access, n	Number PIPAC procedure, n	Median number PIPAC/patient	Median PCI	Acites, n	Bimodal treatment, n	Previous chemotherapy, n
Alyami et al. [9]	Various	73	57.1	0	164	2.6	19	35	64	64
Balmer et al. [6]	Various	183	64	0	517		19	42	48	
De Simone et al. [10]	Various	67	59	4	171	1.8				
Di Giorgio et al. [11]	Gastric	28	50	2	46	1.7	20	8	26	
Di Giorgio et al. [12]	Bilary tract and pancreatic	20	64	1	45	1.7	20	10	11	20
Ellebæk et al. [13]	Colorectal	24	64	0	74	3	10	7	3	22
Ellebæk et al. [14]	Gastric	20	58	0	52	3.2	13	8	9	19
Falkenstein et al. [15]	Bilary tract and pancreatic	11	58	2	17	1.3	20	13	3	10
Graversen et al. [16]	Various	33	59	3	65	1.9			12	32
Graversen et al. [17]	Various	35	65	0	129	3	14	0	5	32
Hübner et al. [18]	Various	42	66	2	91	2.1	10			40
Giger-Pabst et al. [19]	Mesothelioma	29	62	4	74	2.6	19		7	21
Khomyakov et al. [20]	Various	31	52	0	56	1.8	16			7
Khosrawipour et al. [21]	Bilary tract and pancreatic	20	64	0	41	2.1	26			20
Kim et al. [22]	Various	17	62	1	24	1.4	17	11		16
Kurtz et al. [23]	Various	71	58	8	142	2	19	24	42	60
Lurvink et al. [24]	Colorectal	20	64	0	59	3	29	13		12
Nadiradze et al. [25]	Gastric	24	56	1	60	2.4	16		8	19
Rovers et al. [26]	Colorectal	22	64	1	59	3	29	16		12
Sgarbura et al. [27]	Various	101	59	5	251	2.5	19		47	93
Struller et al. [28]	Gastric	25	55.1	0	43	1.7	15	18	0	25
Tempfer et al. [29]	Ovarian	64	62	11	130	2	16	22		
Taibi et al. [30]	Colorectal	131	59	2	343	2.2	20	21	30	127
Tabchouri et al. [31]	Colorectal	102	64	22	185	2.3	14	0	58	99
Sindayigaya et al. [32]	Gastric	144	57	11	216	2	15			131
Tempfer et al. [33]	Gynaecologic	15	60	1	34	2.3				
Total, n (%)		1,352		81	3,088			248	373	881
Median, Q1–Q3 per study		30 (20–70)	60 (58–64)	1 (0–3.7)	69 (47–158)	2.1 (1.8–2.5)	19 (15.1–20)	13 (8–21.2)	11.5 (6.5–43.2)	22 (19–60)
Mean, SD per study		**52 ± 45.5**	**60.2 ± 4.1**	**3.1 ± 5**	**118.8 ± 113.4**	**2.2 ± 0.5**	**18.2 ± 5**	**15.5 ± 11.4**	**23.3 ± 21.8**	**42 ± 38.6**

The median number of PIPAC procedures per patient was 2.1 (1.8–2.6). Only 373 patients (27.6 %) received concomitant chemotherapy (bimodal treatment). The median PCI at the time of the first PIPAC was 19 [15], [16], [17], [18], [19], [20].

In patients who underwent PIPAC, adverse events were graded according to the Common Terminology Criteria for Adverse Events (v4.0 CTCAE) in all studies. The median rate of major postoperative complications (CTCAE grade 3 and 4) was 3.1 % (0.8–6.2 %). Mortality rate was 0.5 % (0–3.9 %) (Table 2).

**Table 2:** PIPAC clinical data summary.

Reference	Number patients at least 1 PIPAC	PIPAC <3, n	PIPAC >3, n	Morbidity (CTCAE 3 ou 4)/PIPAC	Mortality/study
Alyami et al. [9]	73	42	31	9.7 %	6.8 %
Balmer et al. [6]	183	88	95	2.3 %	1.6 %
De Simone et al. [10]	63	43	24	2.4 %	
Di Giorgio et al. [11]	26	17	11	4 %	3.8 %
Di Giorgio et al. [12]	19	13	7	0 %	0 %
Ellebæk et al. [13]	24	9	15	2.7 %	0 %
Ellebæk et al. [14]	20	10	10	3.8 %	0 %
Falkenstein et al. [15]	11	10	1	0 %	
Graversen et al. [16]	30	21	12	0 %	6 %
Graversen et al. [17]	35	8	27	3.9 %	
Hübner et al. [18]	40	24	18	1 %	1 %
Giger-Pabst et al. [19]	25	13	16	4 %	4 %
Khomyakov et al. [20]	31	23	8	3.2 %	0 %
Khosrawipour et al. [21]	20	10	10	0 %	2.4 %
Kim et al. [22]	16	16	1	0 %	0 %
Kurtz et al. [23]	63	43	28	0.7 %	0 %
Lurvink et al. [24]	20	7	13	0 %	0 %
Nadiradze et al. [25]	23	14	10	11.7 %	8.3 %
Rovers et al. [26]	20	8	14	11.9 %	5 %
Sgarbura et al. [27]	101	47	54	15.9 %	0 %
Struller et al. [28]	25	19	6	12 %	0 %
Tempfer et al. [29]	53	19	45	6.9 %	0 %
Taibi et al. [30]	131	72	59	9.3 %	0 %
Tabchouri et al. [31]	80	46	56	3.9 %	0.5 %
Sindayigaya et al. [32]	131	86	58	1.4 %	1.5 %
Tempfer et al. [33]	15	6	9	2.9 %	6.6 %
Total, n	1,278	714	638		
Median (Q1–Q3) per study	28 (20–63)	18 (10–42.7)	14.5 (10–30.2)	3.1 % (0.8–6.2 %)	0.5 % (0–3.9 %)
Mean SD±, per study	49.2 ± 43.8	27.5 ± 24	24.5 ± 23.1	4.4 % ± 4 0.6 %	2.1 % ± 2.7 %

**Figure 2:**
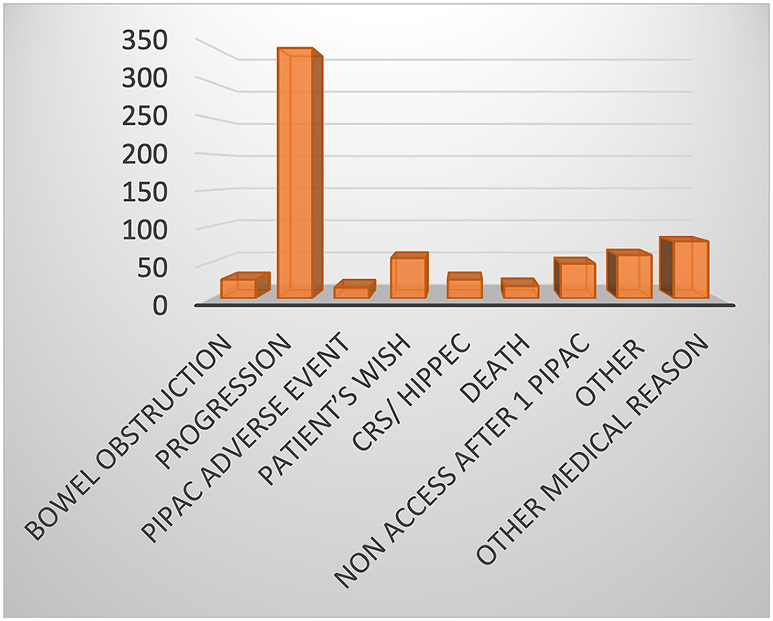
Reason for discontinuing PIPAC treatment.

The number of patients who did not complete the 3 planned PIPAC sessions was 714 (52.8 %) and the median failure of 3 planned PIPAC sessions was about 56.1 % (44.9–62.9 %). After analysing the literature, the main reason for discontinuing PIPAC treatment was disease progression (49.1 %). All reasons for PIPAC interruption are described in Figure 2 and Table 3.

**Table 3:** Reasons for PIPAC interruption before 3 planned PIPAC.

Reference	PIPAC <3, n	Bowel obstruction or perforation, n; %	Progression, n; %	PIPAC adverse event, n; %	Patient’s wish, n; %	CRS/HIPEC, n; %	Death, n; %	Non access after 1 PIPAC, n; %	Other (missing patient), n; %	Other medical reason, n; %
Alyami et al. [9]	42	7; 16.7 %	2; 4.8 %	0 %	0 %	2; 4.8 %	5; 11.9 %	4; 9.5 %	0 %	22; 52.4 %
Balmer et al. [6]	88	13; 14.8 %	44; 50 %	1; 1.1 %	9; 10.2 %	6; 6.8 %	3; 3.4 %	2; 2.3 %	2; 2.3 %	8; 9.1 %
De Simone et al. [10]	43	0 %	43; 100 %	0 %	0 %	0 %	0 %	0 %	0 %	0 %
Di Giorgio et al. [11]	17	0 %	9; 52.9 %	2; 11.8 %	5; 29.4 %	1; 5.9 %	0 %	0 %	0 %	0 %
Di Giorgio et al. [12]	13	0 %	7; 53.8 %	0 %	0 %	0 %	0 %	0 %	1; 7.7 %	5; 38.5 %
Ellebæk et al. [13]	9	1; 11.1 %	2; 22 %	0 %	5; 55.6 %	0 %	1; 11.1 %	0 %	0 %	0 %
Ellebæk et al. [14]	10	0 %	6; 60 %	0 %	0 %	0 %	0 %	0 %	0 %	4; 40 %
Falkenstein et al. [15]	10	2; 20 %	4; 40 %	0 %	0 %	0 %	0 %	0 %	0 %	4; 40 %
Graversen et al. [16]	21	0 %	9; 42.9 %	0 %	3; 14.3 %	0 %	2; 9.5 %	0 %	4; 19 %	3; 14.3 %
Graversen et al. [17]	8	3; 37.5 %	1; 12.5 %	0 %	3; 37.5 %	0 %	1; 12.5 %	0 %	0 %	0 %
Hübner et al. [18]	24	0 %	5; 20.8 %	0 %	3; 12.5 %	0 %	0 %	0 %	1; 4.2 %	Incompleted for 2 PIPAC
Giger-Pabst et al. [19]	13	0 %	6; 46.2 %	0 %	2; 15.4 %	1; 7.7 %	1; 7.7 %	3; 23.1 %	0 %	0 %
Khomyakov et al. [20]	16	0 %	8; 50 %	0 %	0 %	0 %	0 %	0 %	3; 18.8 %	5; 31.3 %
Khosrawipour et al. [21]	10	0 %	7; 70 %	0 %	0 %	0 %	0 %	3; 30 %	0 %	0 %
Kim et al. [22]	16	0 %	5; 31.3 %	0 %	3; 18.8 %	0 %	0 %	0 %	0 %	Incomplead for 2 PIPAC
Kurtz et al. [23]	43	0 %	18; 41.9 %	11; 25.6 %	0 %	0 %	0 %	0 %	0 %	4; 9.3 %
Lurvink et al. [24]	7	0 %	5; 71.4 %	0 %	0 %	0 %	1; 14.3 %	1; 14.3 %	0 %	0 %
Nadiradze et al. [25]	14	0 %	10; 71.4 %	0 %	0 %	0 %	0 %	1; 7.1 %	0 %	3; 21.4 %
Rovers et al. [26]	8	0 %	6; 75 %	0 %	0 %	0 %	0 %	0 %	0 %	2; 25 %
Sgarbura et al. [27]	47	0 %	18; 38.3 %	0 %	2; 4.3 %	3; 6.4 %	0 %	6; 12.8 %	10; 21.3 %	8; 17 %
Struller et al. [28]	19	0 %	12; 63.2 %	0 %	2; 10.5 %	2; 10.5 %	0 %	2; 10.5 %	1; 5.3 %	0 %
Tempfer et al. [29]	19	0 %	19; 100 %	0 %	0 %	0 %	0 %	0 %	0 %	0 %
Taibi et al. [30]	72	0 %	38; 52.8 %	0 %	1; 1.4 %	5; 6.9 %	0 %	2; 2.8 %	17; 23.6 %	9; 12.5 %
Tabchouri et al. [31]	46	0 %	7; 15.2 %	1; 2.2 %	14; 30.4 %	5; 10.9 %	3; 6.5 %	14; 30.4 %	0 %	2; 4.3 %
Sindayigaya et al. [32]	86	0 %	54; 62.8 %	0 %	2; 2.3 %	1; 1.2 %	0 %	8; 9.3 %	21; 24.4 %	0 %
Tempfer et al. [33]	6	0 %	2; 33.3 %	0 %	2; 33.3 %	0 %	0 %	2; 33.3 %	0 %	0 %
Total, n (%)	707	26; 3.7 %	347; 49.1 %	15; 2.1 %	56; 7.9 %	26; 3.7 %	17; 2.4 %	48; 6.8 %	60; 8.5 %	79; 11.2 %
Median (Q1–Q3) per study	16.5 (10–42.7)	0 % (0–0%)	50 % (34.6–63 %)	0 % (0–0%)	19 % (0–15.1 %)	0 % (0–5.6 %)	0 % (0–5.7 %)	0 % (0–10.3 %)	0 % (0–5%)	6.7 % (0–22.3 %)
Mean SD±, per study	27.2 ± 24.1	3.8 ± 9 %	49.3 ± 24.2 %	1.6 ± 5.4 %	10.6 ± 15 %	2.3 ± 3.7 %	3 ± 4.9 %	7.1 ± 10.7 %	4.9 ± 8.5 %	13.1 ± 16.4 %

Other reasons for the premature cessation of planned PIPAC sessions were bowel obstruction, adverse events (problem with healing, toxicity, etc.), patient wishes, curative intent cytoreductive surgery (CRS) and Hyperthermic IntraPEritoneal Chemotherapy (HIPEC), no secondary peritoneal access, medical reasons (embolism, sepsis, etc.) and other reasons (protocol violation, loss of view, no reason found).

We performed a subgroup analysis between studies with a failure rate of 3 planned PIPAC sessions ≥50 % (FAILURE Group) and studies with a failure rate <50 % (SUCCESS Group) (Table 4). The number of studies is small, but notably, in the >50 % FAILURE group, all the treated series include PM from gastric and hepato-biliary tumours. In the SUCCESS group, we observed more patients treated for colorectal cancer or mesothelioma. Statistical analysis showed that patients in the “SUCCESS group” had a higher average mean age (58 vs. 63; p=0.007), but no statistical difference was found in terms of the presence of ascites (p=0.285), bimodal treatment (p=0.263) or previous chemotherapy (p=0.224).

**Table 4:** Comparison between SUCCESS and FAILURE group.

	FAILURE group	SUCCESS group	p-Value
Number of studies	16	10	
Mean age of patients, years	58 ± 4	63 ± 2	0.007
Origin			
Various	7 (43.7 %)	3 (30 %)	–
Colorectal	1 (6.25 %)	4 (40 %)	0.217
Biliary tract and pancreatic	3 (18.75 %)	0	<0.0001
Mesothelioma	0	1 (10 %)	<0.0001
Gastric	5 (31.25 %)	0	<0.0001
Ovarian	0	1 (10 %)	<0.0001
Gynaecologic	0	1 (10 %)	<0.0001
PCI	18 ± 4	19 ± 6	0.951
Ascites presence	52 ± 30 %	32 ± 29 %	0.285
Bimodal treatment	46 ± 29 %	30 ± 18 %	0.263
Previous chemotherapy	88 ± 20 %	80 ± 17 %	0.224

We performed a subgroup analysis by creating a group of studies with >50 % PIPAC discontinuation due to progression and a group with <30 % PIPAC discontinuation due to progression (Table 5). By focusing on cancer types, it appears that colorectal origin causes less progression than cancer of gastric or biliopancreatic origin. Statistical analysis showed that patients in studies stopping PIPAC due to progression in more than 50 % of cases had a higher average PCI (14 ± 4 vs. 20 ± 5 p=0.014), but no statistical difference was found in terms of the presence of ascites (p=0.134), bimodal treatment (p=0.927) or previous chemotherapy (p=0.828). We observed more discontinuation due to progression in cases of gastric cancer.

**Table 5:** Comparison between group with less than 30 % of failure and group with more than 50 % of failure.

	Rate of stop for progression <30 %	Rate of stop for progression ≧50 %	p-Value
Number of studies	5	14	
Mean age of patients, years	63 ± 3	59 ± 4	0.062
Origin			
Various	3 (60 %)	3 (21 %)	
Colorectal	2 (40 %)	3 (21 %)	
Biliary tract and pancreatic	0	2 (14 %)	
Mesothelioma	0	0	
Gastric	0	5 (36 %)	
Ovarian	0	1 (7 %)	
Gynaecologic	0		
PCI median	14 ± 3	19 ± 6.4	0.014
Ascites presence	15 ± 31 %	32 ± 29 %	0.134
Bimodal treatment	36 ± 3 %	30 ± 18 %	0.927
Previous chemotherapy	92 ± 3 %	80 ± 17 %	0.828

## Discussion

Reasons for discontinuing PIPAC before 3 recommended PIPAC sessions are oncological progression, conversion to CRS/HIPEC, patient wishes, adverse events after PIPAC, medical reasons or death [6]. Fewer studies reported a reason for stopping PIPAC, as our review demonstrated; among all the articles >200, only 26 described the reason for discontinuing PIPAC before 3 cycles.

The results of this review of the available literature suggest that early discontinuation of the 3 planned sessions is a problem that is repeatedly reported, with a median number of 2.3 PIPAC sessions. In 2018, Nowacki et al. reported similar results in a multicenter study [7]. Furthermore, the standard treatment protocol for PIPAC consists of 3 procedures and completion of this treatment model has been shown to lead to improved survival and good tumoural response [4, 34, 35]. Our analysis of success rate when performing the 3 planned PIPAC sessions showed that failure and the premature cessation of PIPAC is more frequent in young patients with gastric PM.

The main reason for the interruption of treatment before the 3 planned PIPAC sessions in our review was oncological progression. The mean rate was 49.3 ± 24.2 %. In some series, this rate increases to over 70 % [10, 21, 24], [25], [26, 29] which can be partially explained by the fact that most patients treated with PIPAC are in palliative situations with advanced, aggressive and refractory disease. Discontinuing before 3 planned PIPAC sessions for progression or clinical deterioration raises questions around the selection of patients. The potential indications for PIPAC are PM from colorectal cancer, gastric cancer, ovarian cancer, pancreatic and biliary tract cancer, peritoneal mesothelioma and appendiceal cancer^1^. In our analysis of discontinuation due to progression, in more than 50 % of cases, we found that young patients with a higher PCI stop the PIPAC protocol earlier.

PIPAC therapy also appears to be legitimate for patients who are not eligible for CRS/HIPEC and do not tolerate or have developed systemic chemotherapy intolerance [1]. For many patients, the absence of proof of efficiency for second- or third-line systemic treatments makes PIPAC a very promising option. It is probably this hope that makes us consider this treatment for our patients and overestimate the potential results.

Other reasons for stopping PIPAC before the 3 planned procedures are abdominal non-access; our review showed a rate of 6.8 %. Some studies have reported rates as high as 30 % [21, 31, 33]. After at least one PIPAC session, intraperitoneal chemotherapy and repeated PIPAC are well known causes for the induction of peritoneal sclerosis [36]. Moreover, patients who are eligible for PIPAC treatment often have prior abdominal surgery as CRS or HIPEC. This may explain the observed failure rate which is higher due to attempts to access the abdomen to perform PIPAC [37, 38].

For the treatment of resectable peritoneal metastasis, cytoreductive surgery and HIPEC could be performed for colorectal, ovarian and gastric peritoneal metastasis as well as for pseudomyxoma peritonei and peritoneal mesothelioma. For unresectable PM, systemic chemotherapy and targeted therapy remain the standard of care [1]. Since 2011, PIPAC has been introduced as a novel treatment for PM, with an alternative for patients who are not eligible for CRS and HIPEC [19, 29, 39]. The use of PIPAC as a neoadjuvant treatment has been proposed for different types of cancer [40, 41], with safe and feasible conclusions. Recently, PIPAC demonstrated encouraging results in patients with unresectable PM and Alyami et al. [41] demonstrated that CRS and HIPEC can be achieved in strictly selected patients with unresectable PM at diagnosis after repeated PIPAC. The reason to stop PIPAC had been identified, but with a limited frequency.

Another reason for the discontinuation of the 3 planned PIPAC sessions was patient wishes. The review showed a mean of 7.9 %, but no further explanation was found in the literature. According to Balmer et al. [6] fear of surgery or general anaesthesia and the refusal for repeated hospitalizations are some of the reasons for discontinuing PIPAC.

Bowel obstruction or perforation is some of the reasons for interrupting the 3 planned PIPAC sessions. Bowel obstruction is often a reflection of oncological disease progression with occlusion on carcinosis. Balmer et al. [6] demonstrated that the absence of a prior history of bowel obstruction before the first PIPAC session was associated with the completion of PIPAC treatment.

Post-operative morbidity is a reason for discontinuing PIPAC. Our review showed that adverse events/post-operative complications are reasons for discontinuing PIPAC and found a mean morbidity rate with CTCAE grade 3 and 4 of 8 %. This is in accordance with other reviews of the literature [2, 42], [43], [44]. Recently, Alyami et al. summarised 45 clinical studies with 1,810 PIPAC procedures in 838 patients [1]. The review found that repeated PIPAC sessions were feasible, with a 3 % incidence of post-operative surgical complications. Among adverse events, the most common intraoperative complication was an iatrogenic bowel injury (0–3 % of total PIPAC procedures) [45]. In a previous literature review, Winkler et al. [43] found that bowel injury related to a Veress needle or trocar insertion was the most common complication. For this reason, clinical teams continue to search for improved means of access to the peritoneal cavity to avoid complications and the deterioration of therapeutic management [37].

The main limitations of our study were the missing data for all retrospective studies and the lack of case series describing the motivations to stop PIPAC. Therefore, it would be useful to better describe the reasons of stopping PIPAC in the upcoming studies.

## Conclusions

Many treatment parameters of PIPAC, such as the minimum number of PIPAC applications to induce maximum tumour regression, are still empirical with limited available evidence. It is assumed by most pioneers of PIPAC therapy that at least 3 PIPAC sessions are probably required to induce a substantial regression of PM. Many patients do not complete the 3 planned PIPAC sessions, especially due to rapid oncological progression. This review seemed to demonstrate that PIPAC could be more successful in the case of colorectal, ovarian or mesothelioma PM. It appears that PIPAC in gastric PM gives poor results, especially in young patients with high PCI. As reported by Balmer et al. investigations are necessary to better understand the causes, in order to improve the selection of patients who are most likely to benefit from PIPAC.
